# Comment on “Will HPV vaccination prevent cervical cancer”

**DOI:** 10.1186/s12916-020-01589-8

**Published:** 2020-04-27

**Authors:** Shaokai Zhang, Fanghui Zhao

**Affiliations:** 1 Affiliated Cancer Hospital of Zhengzhou University, Henan Cancer Hospital, Zhengzhou, 450008 China; 2 National Cancer Center/National Clinical Research Center for Cancer/Cancer Hospital, Chinese Academy of Medical Sciences and Peking Union Medical College, Beijing, 100021 China

**Keywords:** Cervical cancer, HPV prophylactic vaccine, Clinical trial

## Background

Cervical cancer is caused by infection with high-risk genotypes of human papillomavirus (HPV). The success of prophylactic HPV vaccine and the rapid development of HPV testing for screening have warranted the bright future of prevention of cervical cancer. In May 2018, the WHO Director-General called for global action to eliminate cervical cancer as a public health problem through improving coverage for HPV vaccination, high-precision screening, and appropriate treatment and care. However, under the circumstance of overwhelming reaction to the WHO call worldwide, a concern on “will HPV vaccination prevent cervical cancer” has been raised.

Rees et al. [1] summarized the data of twelve randomized control clinical trials (RCTs) of Cervarix and Gardasil. Their analysis criticized that these trials generated significant uncertainties undermining claims of efficacy on the basis of the following considerations. Firstly, the sample in a trial cannot certainly represent the vaccination target groups in a real-life setting due to differences in age and restrictive trial inclusion criteria. Secondly, it is still uncertain whether HPV vaccine can prevent cervical cancer as cervical intraepithelial neoplasia (CIN) 2 or worse lesions (CIN2+) were used as surrogate endpoints for this direct outcome. Thirdly, frequent screening in trials could also bias the efficacy and persistent infection evaluation. The above critical appraisal sounds reasonable but beyond the reality in terms of feasibility.

## Target Populations

To the best of our knowledge, the evaluation of each prophylactic HPV vaccine has been conducted in the multi-center, double-blinded, randomized control and population-based prospective clinical trials, and they presented 90–100% protection against cervical persistent infections and CIN2+ linked to HPV 16 and 18 in women aged from 15 up to 45 years who were not infected at vaccination time [2, 3]. The vaccine is designed to prevent incident infection and disease, for which young adolescents before sexual debut are the optimal target group, however, the invasive cervical sample collection limits the clinical efficacy evaluation of HPV vaccine among the adolescents. Subsequently, the immunobridging study was conducted to provide sufficient evidences for protection assessment against adolescents by comparing the serum antibody titers between two groups of adult and adolescent.

## Endpoints

The use of CIN2+ instead of cervical cancer as a clinical endpoint would need a large, labor-intensive, and time-consuming trial because cervical cancer tends to develop less frequently and less rapidly which could hinder the development of vaccines. Meanwhile, it was ethically prohibited in most of the countries. As to the concern of CIN2 could be an imprecise diagnosis, WHO updated the classification of CIN-related lesions, and dichotomy classification was used from 2014. To safely streamline vaccine assessment in the future, the International Agency for Research on Cancer (IARC) recommended HPV persistent infection as the primary endpoint on the basis of experience and the present knowledge of HPV infection and trials. A virological primary endpoint is more reproducibly measured and occurs more frequently than CIN2+. The immunobridging trials can be sufficient to ascertain immunological non-inferiority for licensure of alternate dosing schedules, bridging to age 26 years or younger, and biosimilar vaccines, with post-licensure surveillance confirming effectiveness [4]. Downgrading surrogate marker would enable the trials to be accomplished without imposing a substantial additional cost or time on the development of clinically significant cancer precursors, thereby expediting the introduction of HPV vaccines or optimum use of these effective vaccines.

## Screening Frequency

Another important fact is that the screening visit schedule in trials was more frequent than that in regular screening programs. The 6–12-month screening interval was on the basis of the natural history of HPV persistent infection and the development of CIN2+ to avoid missed lesions. Although CIN2+ could regress spontaneously to some extent, the possibility should be distributed equally between intervention and control arms. For the 6–12-month screening schedule, it could also provide appropriate data for the antibody titers, durability, and virological evaluation which could be helpful to address difficulties in relation to the endpoint [4].

## Real-world Evidence

Although Rees et al.’s review has revealed some methodological limitations with the phase 2 and 3 efficacy trials of HPV vaccination, the results showed strong evidence of the protection of HPV vaccine resulting in the licensure approval in over 160 countries. That said, it is important that the limitations highlighted by Rees et al.’s review will be adequately addressed by long-term follow-up of post-licensure monitoring and real-world studies. For example, the most recent updated systematic review and meta-analysis [5] includes data from 60 million individuals and up to 8 years of post-vaccination follow-up, showing compelling evidence of the substantial impact of HPV vaccination programs on HPV infections and CIN2+ among girls and women. Further, observational data showing the population-level impact of HPV vaccination from the early adopting countries can also be immensely useful. In Scotland, routine vaccination of girls aged 12–13 years with the bivalent HPV vaccine has led to a dramatic reduction in preinvasive cervical disease (89% for CIN3+, 88% for CIN2+) [6]. Similar trends were also observed after quadrivalent HPV vaccination in Australia [7, 8].

## Conclusions

In conclusion, from the public health perspective, HPV vaccine introduction and scale-up should be formed on a sound data basis as well as pragmatic consideration. Up-to-date estimates of the impact and cost-effectiveness of HPV vaccination and screening at national levels are required. Practicable and cost-effective strategies consist of HPV vaccination, and screening would accelerate the progress of cervical cancer elimination globally [9].

## Data Availability

Not applicable.

## Abbreviations

CIN: Cervical intraepithelial neoplasia
HPV: Human papillomavirus
IARC: International Agency for Research on Cancer
RCTs: Randomized control clinical trials
